# A continuous quality improvement intervention to improve the effectiveness of community health workers providing care to mothers and children: a cluster randomised controlled trial in South Africa

**DOI:** 10.1186/s12960-017-0210-7

**Published:** 2017-06-13

**Authors:** Christiane Horwood, Lisa Butler, Pierre Barker, Sifiso Phakathi, Lyn Haskins, Merridy Grant, Ntokozo Mntambo, Nigel Rollins

**Affiliations:** 1 Centre for Rural Health, University of KwaZulu-Natal, Durban, KwaZulu-Natal South Africa; 2 Institute for Collaboration on Health, Intervention and Policy, University of Connecticut, Storrs, CT United States of America; 3 Institute for Healthcare Improvement, Cambridge, MA United States of America; 4 Department of Maternal and Child Health, Gillings School of Global Public Health, University of North Carolina, Chapel Hill, NC United States of America; 5 Department of Maternal, Newborn, Child and Adolescent Health, World Health Organization, Geneva, Switzerland

**Keywords:** Community health worker, Quality improvement, Breastfeeding, South Africa, Maternal health, Child health, HIV

## Abstract

**Background:**

Community health workers (CHWs) play key roles in delivering health programmes in many countries worldwide. CHW programmes can improve coverage of maternal and child health services for the most disadvantaged and remote communities, leading to substantial benefits for mothers and children. However, there is limited evidence of effective mentoring and supervision approaches for CHWs.

**Methods:**

This is a cluster randomised controlled trial to investigate the effectiveness of a continuous quality improvement (CQI) intervention amongst CHWs providing home-based education and support to pregnant women and mothers. Thirty CHW supervisors were randomly allocated to intervention (*n* = 15) and control (*n* = 15) arms. Four CHWs were randomly selected from those routinely supported by each supervisor (*n* = 60 per arm). In the intervention arm, these four CHWs and their supervisor formed a quality improvement team. Intervention CHWs received a 2-week training in WHO Community Case Management followed by CQI mentoring for 12 months (preceded by 3 months lead-in to establish QI processes). Baseline and follow-up surveys were conducted with mothers of infants <12 months old living in households served by participating CHWs.

**Results:**

Interviews were conducted with 736 and 606 mothers at baseline and follow-up respectively; socio-demographic characteristics were similar in both study arms and at each time point.

At follow-up, compared to mothers served by control CHWs, mothers served by intervention CHWs were more likely to have received a CHW visit during pregnancy (75.7 vs 29.0%, *p* < 0.0001) and the postnatal period (72.6 vs 30.3%, *p* < 0.0001). Intervention mothers had higher maternal and child health knowledge scores (49 vs 43%, *p* = 0.02) and reported higher exclusive breastfeeding rates to 6 weeks (76.7 vs 65.1%, *p* = 0.02). HIV-positive mothers served by intervention CHWs were more likely to have disclosed their HIV status to the CHW (78.7 vs 50.0%, *p* = 0.007). Uptake of facility-based interventions were not significantly different.

**Conclusions:**

Improved training and CQI-based mentoring of CHWs can improve quantity and quality of CHW-mother interactions at household level, leading to improvements in mothers’ knowledge and infant feeding practices.

**Trial registration:**

ClinicalTrials.Gov NCT01774136

## Background

Community health workers (CHWs) are generally defined as community members chosen by their community to support or provide health interventions at household level; they are linked to the health system, but have shorter training than professional health workers [1]. Deployment of CHWs can address barriers to preventive and curative care, increasing coverage of key interventions including maternal and child health services, and improve continuity of care during pregnancy and the postnatal period [2]. Together, these can accrue substantial health benefits for communities including mothers and children [3].

In South Africa, where HIV prevalence amongst pregnant women varies between 16.9 and 37.4% across provinces [4], maternal and child mortality remain higher than expected despite evidence-based packages of care being available in primary health care (PHC) facilities [5]. To improve survival, coverage of key interventions must increase, particularly early antenatal care (ANC) attendance (before 22 weeks), postnatal care and infant feeding support, prevention of mother-to-child HIV transmission (PMTCT) interventions and early access to antiretroviral therapy (ART) for mothers living with HIV and their infants.

However, improvements in facility-based care for pregnant women, mothers and children may have limited impact. In South Africa, over half of child deaths occur outside of the health facility [6] largely due to a failure to recognise serious illness amongst children in the home, so that children present late to health facilities. CHW-based interventions may bridge this gap and, in some settings, have substantially reduced maternal and neonatal mortality [7–10]. Elsewhere, however, CHW interventions that improved knowledge and care practices amongst mothers had no effect on maternal and child health outcomes [11]. The implementation and outcome of CHW programmes is dependent not only on appropriate training but also on support and effective supervision that is coordinated with PHC services [2].

In South Africa, most CHWs are recruited and employed by the Department of Health and receive a small stipend. CHWs fulfil a variety of roles in the community including home-based care, adherence support for antiretroviral and TB treatment as well as provision of maternal and child health (MCH) services. Their role in provision of MCH services in households includes counselling about early ANC attendance, identification of danger signs in newborns and support for breastfeeding and is clearly described in the Department of Health policy for community-based maternal, child and newborn care [12].

In this study, we implemented the WHO Community Case Management (CCM) training [13], combined with ongoing mentoring using a continuous quality improvement (CQI) approach [14], to improve and maintain skills of CHWs caring for mothers and children in the community. CCM is based on treatment algorithms developed for Integrated Management of Childhood Illness (IMCI) and broadens access to care by equipping CHWs with skills to support pregnant women and assess and manage sick infants and children in the household [15]. We adapted CCM materials to include interventions to support uptake and delivery of PMTCT and ART services.

CQI is a simple, low-tech approach to the management and supervision of health programmes which has been successfully used to improve PMTCT uptake at facility level in South Africa [16, 17]. The CQI method guides practitioners to better performance by using locally generated data to provide feedback on practices and knowledge. Once gaps in performance are shown, health workers generate, develop and test local solutions and track change in performance to achieve improvement [14, 18].

Here, we report the number of CHW visits and changes in maternal knowledge, household child care practices, care-seeking behaviour and uptake of facility-based maternal and child health interventions by mothers residing in households served by CHWs provided with additional training and supervised using a CQI mentoring approach.

## Methods

### Study design

This is a cluster randomised controlled trial in which 30 CHW supervisors in a high HIV prevalence rural community in South Africa were randomly allocated to intervention (*n* = 15) and control (*n* = 15) arms. From the CHWs routinely managed by each supervisor, four were randomly selected (*n* = 60 per study arm). CHWs in the intervention arm received training in WHO HIV-adapted CCM, formed quality improvement teams (comprising the CHW supervisor and four selected CHWs) and participated in bi-monthly mentoring using CQI methods for 15 months including a 3-month lead-in period. Participants received no monetary or other incentives to participate.

To assess the effect of the intervention on maternal health behaviour, knowledge and infant feeding practices, household surveys were conducted amongst independent samples of mothers of infants aged <12 months old residing in households served by participating CHWs. Surveys were conducted at baseline, prior to implementation of CCM training and CQI mentoring and at follow-up, 15 months following initiation of the intervention.

An independent assessment team conducted the two surveys using a structured questionnaire in the local language (isiZulu). Paper questionnaires were used at baseline, and a tablet-based data collection system was used for the follow-up survey.

### Study setting

The study was conducted in Ugu Health District, KwaZulu-Natal (KZN), South Africa. This district has a population of approximately 700 000 people, predominantly living in rural areas. There are 47 fixed PHC clinics, 14 mobile clinics, three district hospitals and one regional hospital located in the district. CHW supervisors are experienced CHWs selected by the local health authorities, have a minimum grade 12 education and three or more years of experience as a CHW. CHWs provide support to households within geographically distinct catchment areas and visit pregnant women and new mothers regularly during pregnancy and post-delivery. At the time of the study, there were 32 CHW supervisors and 956 CHWs providing services to communities in the district. On average, each participating supervisor supervised 26 CHWs (range 7–59).

### Study participants: CHWs and mothers

All CHW supervisors working in the district and all CHWs with a minimum grade 10 education were eligible for randomisation into the study. Supervisors and CHWs identified through randomisation were subsequently invited to participate.

Mothers were recruited into the surveys if they were aged 18 years or more, had an infant aged less than 12 months of age and were living in a household served by a participating CHW. Mothers were not eligible if they had resided in another community in the 12 months prior to the study. All eligible mothers were invited to participate.

### CHW training

All CHWs had received routine 10-day KZN Department of Health training on community-based care of women and infants prior to study implementation. In the intervention arm, CHW improvement teams received two additional weeks of training on community care of pregnant women and newborns based on WHO CCM materials [19] adapted to include relevant aspects of HIV care. Training included guidelines on antenatal and postnatal visits, with information on HIV and PMTCT, as well as identification of signs of illness in newborn infants and children. Training was supported by detailed materials, conducted in the local language (isiZulu) and included both theoretical and clinical components. Training was conducted with three groups of 25 participants between May and August 2012.

### CQI intervention

The 12-month CQI mentoring intervention was preceded by a 3-month lead-in phase to establish mentoring meetings and processes and was conducted between August 2012 and November 2013. CHWs routinely documented and summarised a limited set of data for women they visited, including gestational age at first booking (from ANC cards), if women had ever tested for HIV (without documenting test results), coverage of PMTCT interventions, delivery site (home or facility) and early infant feeding practices. CHW improvement teams received bi-monthly mentoring from experienced quality mentors based at the University of KZN and employed by the project, using a CQI approach. During mentoring meetings, teams reviewed data elements collected by CHWs to identify gaps and together design tests of ideas for how challenges could be resolved. Quarterly learning sessions were convened at which CHW improvement teams presented their progress to provide opportunities for peer learning across CHW groups. This intervention is described further elsewhere [20].

### Measurements at baseline and follow-up

#### Care-seeking

Mothers were asked if she had needed to go to the clinic in the past 6 months, either for herself or for her child, but had not gone for any reason.

#### CHW visits

Mothers were asked if and how often the CHW had visited during pregnancy and after delivery and if the CHW had provided information about key antenatal and postnatal health topics.

#### Maternal, neonatal and child health knowledge

Knowledge was assessed using four questions. A total knowledge score for each participant was calculated; see Table 1 for details on scoring.

**Table 1:** Scoring system for maternal knowledge

Question	Correct answer(s) and coding	Max
Q1. When in your pregnancy should you go for your first antenatal visit?	*Correct answer:* As soon as you have missed your period or as soon as you suspect you are pregnant or similar Score = 1	1
Q2. How many antenatal visits should you go for during your pregnancy?	*Correct answer:* four or more Score = 1	1
Q3. What are danger signs you should look out for during your pregnancy?	*Correct answers:* Vaginal bleeding Shortness of breath Severe abdominal pain Fits Headaches Swelling of feet and face Decreased movements of the baby Score = no. of correctly mentioned	7
Q4. What are the danger signs that would make you take a newborn baby urgently to the clinic?	*Correct answers:* Not able to feed well Had a convulsion or fit Shortness of breath High temperature Low temperature Yellow skin and eyes Only moves when stimulated Pus draining from umbilicus Pus draining from eyes Score = no. of correctly mentioned	8
Total knowledge score	Mean score of Q1 + Q2 + Q3 + Q4	17

#### Infant feeding practices

Mothers of infants under 6 months old were asked if they had ever breastfed their infant, if they were currently breastfeeding and, if the infant was >6 weeks old, how they fed their infant during the first 6 weeks of life.

### Statistical analysis

Demographic characteristics in baseline and follow-up study samples in the intervention and control groups were summarised by percentages, means and standard deviations. Outcomes were compared between intervention and control groups using intention to treat analysis. Outcomes were compared at each time point and changes in each group compared between time points. These comparisons were made first without adjustment, followed by repeated-measures linear or logistic regression, as appropriate, for continuous or dichotomous measures, respectively, with random intercepts for supervisors and CHWs within the experimental group. The regression models used change between time points as the dependent variable. To account for within-subject correlation of measurements over time, we used the SAS MIXED procedure for numeric outcomes [21] and SAS GENMOD procedure to implement the generalised estimating equation (GEE) approach for binary outcomes [22, 23]. Results were expressed as odds ratios (OR) with 95% CI. The *p* values were two-sided and statistical significance was defined at the 5% level. All analyses were conducted with type I error set at 0.05 for each pairing of dependent and independent variables. The SAS statistical software package 9.2 was used for all computations.

### Ethics

Written informed consent was obtained from all participating CHWs, supervisors and mothers. Ethical approval was obtained from the Biomedical Ethics Review Committee at the University of KwaZulu-Natal (BGC077/11), World Health Organization and the University of California, San Francisco (12-08526). Permission to undertake the study was obtained from the KZN Department of Health.

## Results

**Fig 1:**
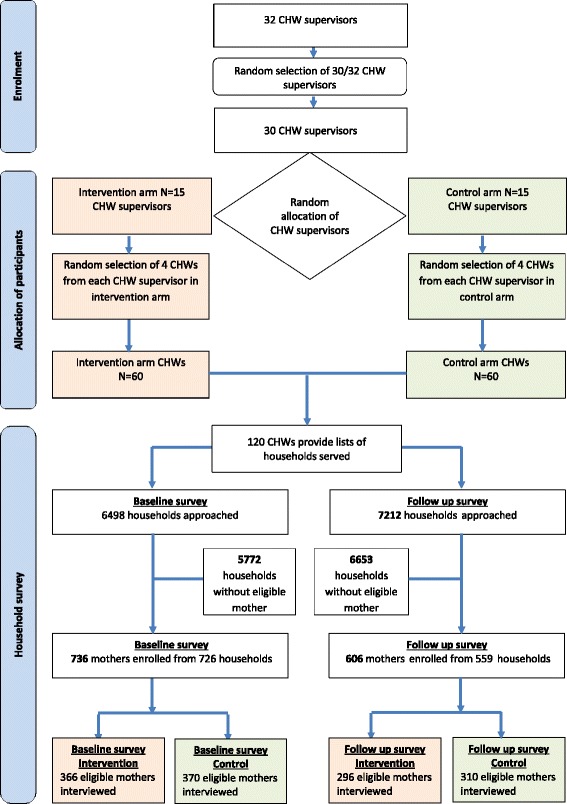
Recruitment and allocation of participants

The baseline survey was conducted between April and August 2012 and the follow-up survey between November 2013 and March 2014. Participating CHWs provided a list of households served at each time point. One intervention CHW dropped out due to language difficulties and one control CHW died during the course of the study; however, data was collected both at baseline and follow-up in the households served by these participants. Each CHW served an average of 62 households (range 28–155). Participant enrolment and allocation are shown in Fig. 1.

Social and demographic characteristics of interviewed mothers are described in Table 2. These were similar in the intervention and control arms at both survey time points.

**Table 2:** Characteristics of mothers who participated in household surveys at baseline, prior to intervention and 12 months after the intervention

	Baseline household survey T1			Follow-up household survey T2		
	Control *N* = 370	Intervention *N* = 366	Total *N* = 736	Control *N* = 310	Intervention *N* = 296	Total *N* = 606
Mother’s age (median, IQR)	25.0 (21.0–30.0)	24.0 (20.0–30.0)	24 (21.0–30.0)	23.0 (20.0–29.0)	23.0 (20.0–28.0)	23.0 (20.0–29.0)
	*p* value = 0.63			*p* value = 0.65		
Youngest child’s age (months) (median, IQR)	5.5 (2.5–8.6)	5.2 (2.7–8.9)	5.4 (2.6–8.7)	6.1 (3.0–8.0)	6.0 (2.8–8.8)	6.0 (2.9–8.8)
	*p* value = 0.80			*p* value = 0.98		
Education						
None	6 (1.6)	8 (2.2)	14 (1.9)	5 (1.6)	6 (2.1)	11 (1.8)
Grade 1 to Grade 8	74 (20.1)	82 (22.7)	156 (21.3)	49 (15.9)	64 (22.1)	113 (18.9)
Grade 9 to Grade 12	286 (77.5)	267 (73.8)	553 (75.7)	240 (77.7)	215 (74.1)	455 (76.0)
Post-school qualification	3 (0.81)	5 (1.4)	8 (1.1)	15 (4.9)	5 (1.7)	20 (3.3)
	*p* value = 0.65			*p* value = 0.09		
HIV serostatus						
HIV-positive	111 (30.0%)	99 (27.1%)	210 (28.5%)	84 (27.1%)	80 (27.0%)	164 (27.1%)
HIV-negative	257 (69.5%)	260 (71.0%)	517 (70.2%)	218 (70.3%)	200 (67.6%)	418 (69.0%)
Do not know or refused to answer	2 (0.54%)	7 (1.9%)	9 (1.2%)	8 (2.6%)	16 (5.4%)	24 (4.0%)
	*p* value = 0.89			*p* value = 0.45		
Employed	32 (8.7)	28 (7.8)	60 (8.2)	25 (8.1)	22 (7.5)	47 (7.8)
	*p* value = 0.67			*p* value = 0.83		
Received child support grant	281 (76.0)	260 (71.4)	541 (73.7)	221 (71.8)	192 (66.0)	413 (69.0)
	*p* value = 0.46			*p* value = 0.17		
Father of child alive	360 (97.3)	357 (97.8)	717 (97.6)	306 (99.4)	288 (99.0)	594 (99.2)
	*p* value = 0.95			*p* value = 0.43		
Father of child lives in home (if alive)	68 (18.9)	86 (24.1)	154 (21.5)	48 (15.7)	52 (18.1)	100 (16.8)
	*p* value = 0.13			*p* value = 0.47		
Adult household member died in past 12 months	50 (13.7)	45 (12.5)	95 (13.1)	57 (18.5)	53 (18.2)	110 (18.4)
	*p* value = 0.69			*p* value = 0.96		
Time to clinic (hours) (median, IQR)	0.50 (0.33–1.0)	0.50 (0.42–1.0)	0.50 (0.42–1.0)	0.50 (0.33–1.0)	0.50 (0.46–1.0)	0.50 (0.33–1.0)
	*p* value = 0.33			*p* value = 0.56		
Cost of transport to clinic (South African rand) (median, IQR)	5 (0–9)	7 (0–9)	6.9 (0–9)	6 (0–10)	6 (0–10)	6 (0–10)
	*p* value = 0.72			*p* value = 0.54		

### CHW visits

The frequency of and activities completed during CHW visits amongst pregnant women are shown in Table 3. Improvements in mother-reported provision of health information were observed amongst intervention CHWs compared to control CHWs. Changes differed significantly between groups (time × treatment interaction) with respect to provision of information about early ANC attendance (*p* < 0.0001), danger signs in pregnancy (*p* < 0.0001), importance of going to clinic for postnatal care (*p* < 0.0001), infant feeding (*p* < 0.0001) and danger signs in newborn babies (*p* < 0.0001). Provision of breastfeeding assistance was higher in the intervention group (*p* < 0.001).

**Table 3:** Community health worker activities as reported by participating mothers at baseline and follow-up household surveys

	Baseline household survey		Follow-up household survey		Change—I-C
	*N* (%)	OR (95% CI)	*N* (%)	OR (95% CI)	OR (95% CI)
CHW visited during pregnancy					
Control	111/370 (30.0%)	Reference	90/310 (29.0%)	Reference	
Intervention	79/366 (21.6%)	OR = 0.64 (0.35–1.2) *p* value = 0.15	224/296 (75.7%)	8.3 (4.4–15.6) *p* value <0.0001	13.0 (7.6–22.2), *p* value <0.0001
CHW provided information about attending antenatal care as early as possible in pregnancy					
Control	95/370 (25.7%)	Reference	76/310 (24.5%)	Reference	
Intervention	59/366 (16.1%)	OR = 0.56 (0.29–1.1) *p* value = 0.09	217/296 (73.3%)	OR = 9.9 (5.0–19.6) *p* value < 0.0001	OR = 17.6 (9.9–31.3) *p* value < 0.0001
CHW provided information about danger signs in pregnancy					
Control	86/370 (23.2%)	Reference	70/310 (22.6%)	Reference	
Intervention	54/366 (14.8%)	OR = 0.61 (0.30–1.2) *p* value = 0.16	214/296 (72.3%)	OR = 9.9 (4.9–19.9) *p* value <0.0001	OR = 16.4 (9.2–29.3) *p* value <0.0001
CHW provided information about going to the clinic for postnatal care					
Control	91/370 (24.6%)	Reference	76/310 (24.5%)	Reference	
Intervention	62/366 (16.9%)	OR = 0.63 (0.32–1.2) *p* value = 0.19	216/296 (73.0%)	OR = 9.5 (4.8–19.0) *p* value <0.0001	OR = 15.1 (8.5–26.7) *p* value <0.0001
CHW provided information about how to feed baby after birth					
Control	92/370 (24.9%)	Reference	74/310 (23.9%)	Reference	
Intervention	53/366 (14.5%)	OR = 0.52 (0.26–1.0) *p* value = 0.06	211/296 (71.3%)	OR = 8.9 (4.5–17.6) *p* value <0.0001	OR = 17.1 (9.7–30.4) *p* value <0.0001
CHW visited in first month after child was born					
Control	104/370 (28.1%)	Reference	94/310 (30.3%)	Reference	
Intervention	90/366 (24.6%)	OR = 0.83 (0.47–1.5) *p* value = 0.53	215/296 (72.6%)	OR = 6.5 (3.6–11.8) *p* value <0.0001	OR = 7.8 (4.6–13.3) *p* value <0.0001
CHW helped with breastfeeding					
Control	84/370 (22.7%)	Reference	74/310 (23.9%)	Reference	
Intervention	65/366 (17.8%)	OR = 0.74 (0.40–1.4) *p* value = 0.35	194/296 (65.5%)	OR = 6.7 (3.6–12.6) *p* value <0.0001	OR = 9.0 (5.2–15.8) *p* value <0.0001
CHW provided information about danger signs in newborn babies					
Control	83/369 (22.5%)	Reference	65/310 (21.0%)	Reference	
Intervention	49/365 (13.4%)	OR = 0.55 (0.29–1.1) *p* value = 0.08	175/296 (59.1%)	OR = 6.1 (3.2–11.5) *p* value <0.0001	OR = 11.0 (6.2–19.5) *p* value <0.0001

Among only those mothers who reported receiving a visit from a CHW at follow-up, mothers in the intervention group were more likely than control mothers to have received key health information during these visits: 70/90 (77.8%) vs 214/224 (95.5%, *p* = 0.0003) for danger signs in pregnancy, 74/90 (82.2%) vs 211/224 (94.2%, *p* = 0.01) for feeding advice, 65/94 (69.2%) vs 175/215 (81.4%, *p* = 0.01) for danger signs in newborns and 74/94 (78.7%) vs 194/215 (90.2%, *p* = 0.01) received assistance with breastfeeding.

Amongst mothers who self-reported being HIV-positive and being visited by a CHW, 24/43 (55.8%) mothers in the control group and 17/30 (56.7%) mothers in the intervention group reported having disclosed their HIV-positive status to their CHW (*p* = 0.94) at baseline. However, at follow-up, significantly more HIV-positive mothers in the intervention group reported having disclosed to their CHW than mothers in the control group (59/72 [81.9%] vs 23/46 [50.0%] *p* = 0.007).

### Care-seeking

Self-reported care-seeking practices over the preceding 6 months amongst participating mothers at baseline and follow-up are shown in Table 4. At follow-up, significantly fewer intervention mothers reported failing to seeking treatment when needed for themselves or for their child(ren). Time and costs to attend the clinic were similar in both groups at both time points (Table 2).

**Table 4:** Care-seeking practices amongst participating mothers at baseline and follow-up household surveys household survey T1 and T2

	Baseline household survey		Follow-up household survey		Change—I-C, T1–T2
	*N* (%)	OR (95% CI)	*N* (%)	OR (95% CI)	OR (95% CI)
In past 6 months failed to go to the clinic for any reason					
Control	92/370 (24.9%)	Reference	97/310 (31.3%)	Reference	
Intervention	73/366 (20.0%)	0.71 (0.43–1.1), *p* value = 0.16	64/296 (21.6%)	0.55 (0.33–0.90), *p* value = 0.02	0.77 (0.46–1.3), *p* value = 0.34
In past 6 months, your child/ren needed to go to clinic but failed to go for any reason					
Control	78/370 (21.1%)	Reference	76/310 (24.5%)	Reference	
Intervention	65/366 (17.8%)	0.78 (0.47–1.3), *p* value = 0.33	46/296 (15.5%)	0.53 (0.31–0.91), *p* value = 0.02	0.68 (0.38–1.2), *p* value = 0.18

### Infant feeding

Infant feeding practices amongst participating mothers at baseline and follow-up are shown in Table 5. At follow-up, a significantly greater proportion of mothers in the intervention group reported having exclusively breastfed their infant for 6 weeks (*p* = 0.02). Further, the changes from baseline to follow-up differed between groups (*p* = 0.001 for time × treatment interaction).

**Table 5:** Reported infant feeding practices amongst participating mothers at baseline and follow-up household surveys

	Baseline household survey		Follow-up household survey		Change—I-C, T1–T2
	*N* (%)	OR (95% CI)	*N* (%)	OR (95% CI)	OR (95% CI)
Amongst mothers of infants >6 weeks:					
Exclusive breastfeeding for first 6 weeks of life					
Control	226/312 (72.4)	Reference	181/278 (65.1)	Reference	
Intervention	207/317 (65.3)	0.74 (0.49–1.1), *p* value = 0.13	194/253 (76.7)	1.7 (1.1–2.7), *p* value = 0.02	2.3 (1.4–4.0), *p* value = 0.001
Amongst mothers of infants <6 months:					
Ever breastfed infant					
Control	166/199 (83.4)	Reference	123/151 (81.5)	Reference	Reference
Intervention	164/198 (82.8)	0.96 (0.54–1.7), *p* value = 0.87	128/144 (88.9)	1.7 (0.86–3.5), *p* value = 0.12	1.8 (0.78–4.3), *p* value = 0.17

### Knowledge about maternal, neonatal and child health

Knowledge was similar in both intervention and control groups at baseline (mean score 48% in both intervention and control groups, *p* = 0.85). At follow-up, there was a statistically significant difference in mean knowledge scores between intervention and control participants (mean scores 49 vs 43%, *p* = 0.008). When the change in knowledge was examined, there was a statistically significant difference between the two groups (*p* = 0.002 for time × treatment interaction).

### Coverage of facility-based services

Attendance at clinics was high for both groups at all measured times in the study with no significant differences observed. ANC attendance at baseline was high in intervention and follow-up groups (98.6 vs 98.1%, *p* = 0.58) and was similar at follow-up (95.8 vs 98.4%, *p* = 0.09). Attendance for postnatal care within 1 week of delivery was high amongst both intervention and control groups at baseline (87.1 vs 82.8%, *p* = 0.24) and at follow-up (91.7 vs 88.3%, *p* = 0.19), with no differences between groups or at different survey time points. Amongst HIV-positive women at both survey time points, coverage of HIV testing and care and treatment services were high. The proportion of HIV positive mothers currently on ART was high in both intervention and control groups at baseline (87.8 vs 90.4%, *p* = 0.99) and was 98.4% for both intervention and control groups at follow-up (*p* = 0.98).

## Discussion

We found that providing WHO adapted CCM training to CHWs supported by a CQI-based model of mentoring substantially increased the number of antenatal and postnatal household visits by CHWs and the proportion of mothers who were able to recall key health promotion messages. This was associated with improved infant feeding practices and care-seeking behaviours of mothers.

At follow-up, fewer mothers served by intervention CHWs reported failing to seek care if they or their infants were unwell. This may have been due to improved knowledge of mothers regarding danger signs amongst newborns and self-initiated attendance or because of direct prompting by CHWs who were visiting households more frequently.

Mothers served by intervention CHWs were more likely to disclose their HIV status to the CHW. This improved willingness of mothers to disclose their HIV status suggests that relationships and trust between the mother and her CHW improved in the intervention group. Disclosure of HIV status by mothers to CHWs not only creates additional opportunities for CHWs to provide support and advice to mothers living with HIV and their infants but also requires additional training and skills development.

Reported rates of exclusive breastfeeding for the first 6 weeks of life were higher amongst mothers and infants cared for by CHWs receiving the CQI intervention. This was associated with substantially greater interactions and support by CHWs at home to support optimal early infant feeding practices and improved knowledge about breastfeeding amongst mothers. In particular, there was a substantial increase in the proportion of mothers who received help with breastfeeding from the CHW. It appears that mothers were responsive to this support and, whilst exclusive breastfeeding rates were assessed only at 6 weeks of age, it is plausible that feeding practices improved throughout the first year of life. Improving coverage and duration of exclusive breastfeeding is cited as the most effective intervention to save infant lives and improve child health [24]; our findings highlight the significant role that CHWs may have in support of maternal and infant health. In contrast to facility-based interventions where mothers rely on health workers to deliver a specific service, it appears that mothers supported by CHWs were more likely to take the initiative to improve health outcomes such as through health-seeking behaviour and infant feeding practices.

Although we were unable to demonstrate any change in coverage of facility-based interventions, this is likely because antenatal and postnatal attendance rates were already very high in both control and intervention groups at baseline and there was little scope to improve these. Coverage of HIV interventions was also very high at baseline and did not significantly change during the study period.

Although intervention CHWs received additional training based on the WHO CCM modules [19], it is unlikely that this training alone could account for the observed changes in the intervention arm. Training alone is not likely to change health worker performance [25]. Thus, it is likely that the CQI intervention contributed to the improved outcomes shown in this study which were sustained for more than 1 year following training. Supervision and mentoring of health workers is cited as being essential for improving the quality of service delivery [24–27]. In the same way that WHO recommends training, WHO also considers post-training supervision as integral to IMCI, CCM and the management of children with severe acute malnutrition. However, regular and sustained quality supervision and support for trained health workers is uncommon in health services in both low and middle resource settings [28, 29].

CHWs work independently in isolated areas with less formal training than facility-based health workers, and supervision and support is therefore a considerable challenge for health systems. Yet supervision may not only improve performance of CHWs but also raise awareness of their role, legitimise their role in the community and improve CHW motivation and retention [30, 31]. However, it is unclear which supervision model would be most effective in most settings [28], and there is little robust evidence of effectiveness of supervision models for CHWs. This study implemented a CQI-based mentoring approach amongst CHWs performing their routine work and tasks. CHWs collected specific data elements when visiting families and used these on a regular basis with their supervisor to review performance and outcomes; these were also shared at the joint learning sessions. In the past, CQI approaches have generally been used in health facilities with professional staff; in this study, we adapted such approaches so that CHWs, who had limited literacy and numeracy skills, could still utilise the data-driven method. Our study does not compare whether CQI is more or equally effective than other forms of supervision to improve CHW performance; however, we show that CQI can be successfully adapted for use with this cadre of health worker and performance can be improved.

A central consideration of any supervision approach in low resource settings must be scalability and sustainability. In our setting, there were relatively large numbers of CHWs compared to supervisors, making the implementation of any supervision framework challenging. In the study, CHWs and supervisors formed small improvement teams that met regularly. Teams also met with other teams to facilitate peer-to-peer learning and problem-solving. All participants commented on the high acceptability and value they attributed to the CQI approach (data not shown). CQI approaches have been successfully implemented in a number of developing country settings, including some community settings [20]. This can be effective and provides a logical supervision and monitoring framework that can link services in facilities with activities in the community including amongst CHWs.

There are important limitations for scaling up the CQI approach used in this proof of concept study. This approach is resource intensive and requires skilled facilitators and tools to support CHWs data collection, and CHWs have to travel to meetings. However, routine supervision meetings do occur and CHWs collect routine data. These processes could be adapted to CQI principles and the CQI approach could provide the strong framework needed to strengthen supervision. However, this would require buy-in and resource allocation from the Department of Health to develop the required systems and will be particularly challenging given differences in CHW programme administration across districts and provinces. In addition, further evaluation would be required to assess the feasibility and effectiveness of this approach at scale.

## Conclusions

Health systems in southern Africa are commonly under-resourced and frequently inaccessible to the poorest and most isolated in the community. CHWs are an important resource for improving the care of mothers and children in communities and for reaching universal coverage of interventions; however, their effectiveness will be constrained unless adequately trained and supported. Failing to invest the additional resources to provide effective supervision, beyond the human resource costs and supplies, may be a false economy.

## Abbreviations

ANC: Antenatal care
ART: Antiretroviral therapy
CCM: Community case management
CHW: Community health worker
CQI: Continuous quality improvement
HIV: Human immunodeficiency virus
IMCI: Integrated Management of Childhood Illness
IQR: Interquartile range
KZN: KwaZulu-Natal
OR: Odds ratio
PHC: Primary health care
PMTCT: Prevention of mother-to-child transmission
WHO: World Health Organization
